# Designing Nurse–Physician Collaboration to Improve Psychological Safety, Satisfaction and Commitment of Critical Care Nurses—A Multi‐Informant Survey Study

**DOI:** 10.1111/nicc.70567

**Published:** 2026-07-01

**Authors:** Daniel O. Thomas‐Rüddel, Frank Bloos, Hendrik Rüddel, Daniel Schwarzkopf

**Affiliations:** ^1^ Department of Anesthesiology and Intensive Care Medicine Jena University Hospital Jena Germany; ^2^ Center for Sepsis Control and Care Jena University Hospital Jena Germany; ^3^ Department of Anesthesiology Montefiore Medical Center, Albert Einstein College of Medicine Bronx New York USA

**Keywords:** critical care nursing, interprofessional relations, job satisfaction, nurse–physician collaboration, psychological safety

## Abstract

**Background:**

Psychological safety is a key component of effective healthcare teams and may impact job satisfaction, commitment and change readiness among critical care nurses. The design of nurse–physician collaboration may influence psychological safety but has not been systematically studied in intensive care settings.

**Aims:**

To investigate how nurse autonomy, participation in ward rounds, quality of nurse–physician relations and team size relate to psychological safety in critical care nurses, and how psychological safety mediates statistical effects on job satisfaction, affective commitment and commitment to change.

**Study Design:**

We performed a multicentre, cross‐sectional, multi‐informant survey study conducted between December 2013 and March 2015 using a convenience sample of 22 intensive care units (ICUs) in 19 German hospitals, including university, public and private hospitals. Organisational data were provided by ICU leaders. Validated scales were used to assess psychological safety, job satisfaction, affective commitment to unit and commitment to change (nurse survey), and nurse–physician collaboration (physician survey). An organisational questionnaire was developed to assess nurses' autonomy and structural factors. Linear regression and mediation analyses tested hypothesised associations at the unit level.

**Results:**

Of 1216 invited nurses and 379 invited physicians, 600 and 217 participated, respectively. Higher nurse autonomy (0.51 [95% CI: 0.1, 0.91], *p* = 0.016), consistent participation in ward rounds (0.51 [0.11, 0.91], *p* = 0.014), better nurse–physician collaboration (0.59 [0.21, 0.97], *p* = 0.004) and lower number of nurses working in a unit (−0.55 [−0.94, −0.16], *p* = 0.008) were statistically significantly associated with higher psychological safety in linear regression analysis. Psychological safety mediated effects of all predictors on job satisfaction, affective commitment and commitment to change.

**Conclusions:**

Psychological safety among critical care nurses can be enhanced through interprofessional collaboration, nurse autonomy and smaller team structures. These findings inform organisational strategies to improve nurse wellbeing and retention, as well as organisational readiness to change.

**Relevance to Clinical Practice:**

Fostering interprofessional collaboration and enhancing nurses' autonomy in critical care are key to enhancing psychological safety and thereby enhancing job satisfaction and retention. Increasing team size can have unintended negative consequences that need to be weighted against potential gains.

**Trial Registration:** German Clinical Trial Register: DRKS00005357

## Impact Statements

- What is known about the topic
  ○ Psychological safety improves team performance and staff retention.
  ○ ICU nurses often report lower psychological safety than physicians.
- What this paper adds
  ○ ICU nurses feel safer with more autonomy, smaller teams and joint ward rounds.
  ○ Psychological safety mediates positive effects on satisfaction and commitment.

## 1 Background

Nurses make up the largest group of the interprofessional care teams staffing Intensive Care Units (ICUs) [1, 2]. ICUs, alongside operating rooms, are the hospital areas with the highest frequency of diagnostic and therapeutic procedures, including mechanical ventilation and organ support. In addition, there is constant pressure to manage patient flow of planned and unplanned admissions with limited capacity [3]. Interprofessional describes care provided by a team of healthcare professionals with overlapping expertise and an appreciation for the unique contribution of other team members, working as partners in achieving a common goal. Care is provided across disciplinary lines and roles become shared to some extent [2].

The concept of psychological safety as an important determinant of team performance has gained attention in recent years [4, 5]. Common definitions of psychological safety include ‘feeling able to show and employ one's self without fear of negative consequences to self‐image, status, or career’ and ‘a shared belief that the team is safe for interpersonal risk‐taking’ [6]. It is supposed to improve safety culture, quality of care and interprofessional cooperation in healthcare teams [6, 7]. In addition, it is associated with reduced staff turnover [8, 9, 10]. Due to their status in the professional hierarchy, psychological safety on average is lower in nurses compared to physicians [7].

In Germany, due to legal requirements [11] and tradition, healthcare delivery is mostly physician centred. Nonetheless, in the interprofessional ICU setting, nurses take responsibility for many tasks typically seen as ‘a doctor's job’ [1, 12]. However, there is no general agreement or common practice about what tasks should be performed by nurses independently or under physician guidance in the ICU. Instead, there is a high variability in nurses' responsibilities across ICUs [1, 12]. This gives ideal conditions to assess the influence of nurse autonomy in performing ‘physician‐tasks’ on psychological safety, which has not been investigated yet.

## 2 Aims and Research Hypotheses

The aim of this study was to assess the influence of the design of nurse–physician collaboration on psychological safety in German critical care nurses and its association with job satisfaction and organisational commitment.

Based on important theoretical frameworks of organisational science and a review of the literature, three aspects of nurse–physician collaboration as well as the influence of team size were investigated. We developed the following research hypotheses:

Hypothesis 1 *Autonomy and responsibility of nurses as reported by the leading attending/medical director of an ICU is positively associated with psychological safety*.

Hypothesis 2 *Nurses working in an ICU where nurses always participate in ward rounds feel more psychologically safe*.

Hypothesis 3 *Quality of nurse–physician relations and collaboration as reported by physicians is positively associated with psychological safety in nurses*.

Hypothesis 4a *Nurses working in an ICU with a smaller number of nurses working in the unit feel more psychologically safe than nurses working in an ICU with a large number of nurses*.

Hypothesis 4b *The effect of team size on psychological safety is partly mediated by the quality of nurse–physician relations*.

Hypothesis 5.1 *Psychological safety mediates* *a positive effect of nurses' autonomy in patient care on (a) job satisfaction, (b) affective commitment to the unit and (c) commitment to change*.

Hypothesis 5.2 *Psychological safety mediates a positive effect of nurses' participation in ward rounds on (a) job satisfaction, (b) affective commitment to the unit and (c) commitment to change*.

Hypothesis 5.3 *Psychological safety mediates a positive effect of the quality of nurse–physician relations on (a) job satisfaction, (b) affective commitment to the unit and (c) commitment to change*.

Hypothesis 5.4 *Psychological safety mediates the negative effect of unit size on (a) job satisfaction, (b) affective commitment to the unit and (c) commitment to change*.

**FIGURE 1:**
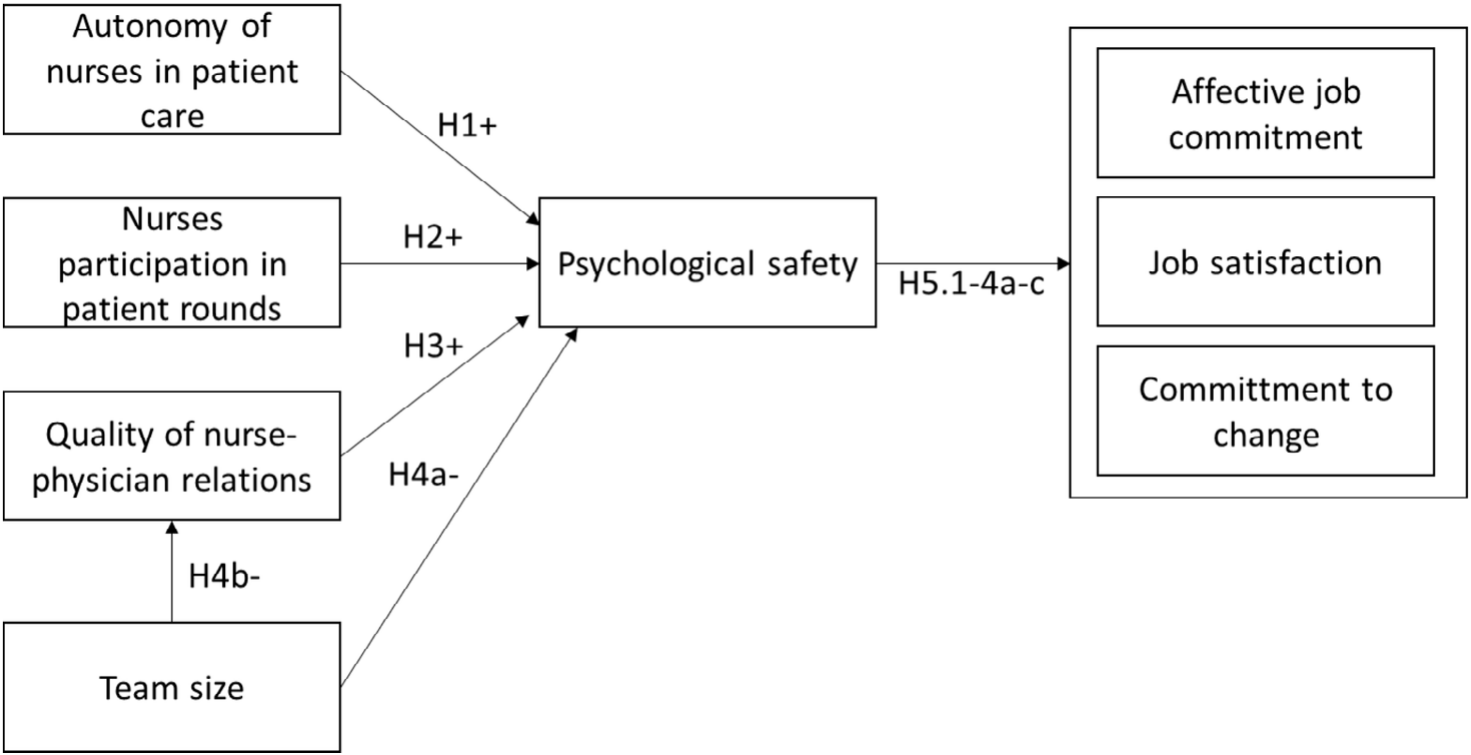
Hypothesised model: Hypothesis 1: Autonomy and responsibility of nurses as reported by the leading attending is positively associated with psychological safety. Hypothesis 2: Nurses working in an ICU where nurses always participate in ward rounds feel more psychologically safe. Hypothesis 3: Quality of nurse–physician relations and collaboration as reported by physicians is positively associated with psychological safety. Hypothesis 4a: Nurses working in an ICU with a smaller number of nurses working in the unit feel more psychologically safe than nurses working in an ICU with a large number of nurses. Hypothesis 4b: The effect of team size on psychological safety is partly mediated by the quality of nurse–physician relations. Hypothesis 5.1: Psychological safety mediates a positive effect of nurses' autonomy in patient care on (a) job satisfaction, (b) affective commitment to the unit and (c) commitment to change. Hypothesis 5.2: Psychological safety mediates a positive effect of nurses' participation in WR on (a) job satisfaction, (b) affective commitment to the unit and (c) commitment to change. Hypothesis 5.3: Psychological safety mediates a positive effect of the quality of nurse–physician relations on (a) job satisfaction, (b) affective commitment to the unit and (c) commitment to change. Hypothesis 5.4: Psychological safety mediates the negative effect of unit size on (a) job satisfaction, (b) affective commitment to the unit and (c) commitment to change.

The complete hypothesised research model is given in Figure 1. Details on the hypothesis development are given in Supporting Information A.

## 3 Design and Methods

### 3.1 Design and Setting

A multi‐centre, cross sectional, standardised survey of ICU nurses and physicians was conducted in a convenience sample of ICUs. Sixteen of 39 invited hospitals participated with a total of 22 intensive care units (ICUs). Besides the topics reported in this manuscript, the survey was also designed to investigate the experience of nonbeneficial treatment and burnout of ICU staff [13] and in comparison with palliative care staff [14], on which results were already published. The study was registered at the German Clinical Trial Register (www.drks.de, DRKS00005357, first registered 10 October 2013). The manuscript was written following the CROSS‐guideline [15]. Methodological details are presented in Supporting Information A.

### 3.2 Procedure and Sample

Experienced senior physicians or heads of department served as local study coordinators (one per unit). All of them answered a centre questionnaire on organisational characteristics of the hospital and the ICU (organisational questionnaire). The local study coordinators also distributed the paper–pencil surveys to ICU staff (staff questionnaire). Data collection took place between December 2013 and March 2015. The study was approved by the institutional review board of the authors' institution as well as all local ethics committees and works councils responsible for the participating centres. Research was conducted in accordance with the Ethical Principles of the German Psychological Society (DGP) and the Association of German Professional Psychologists (BDP) in their most recent edition. Participation in the survey was completely deliberate. Participants received written information on the aims of the study and the confidentiality of their individual answers. As approved by the ethics committee, no written informed consent was collected, but informed consent was implied if participants returned the completed questionnaire. All physicians and nurses working on the ICU at the time of the survey were eligible for participation. Only nurses working at the bedside were included in the analyses; therefore, nurses who identified themselves as head nurses in the survey were excluded.

### 3.3 Ethics Approval and Consent to Participate

The study was approved by the institutional review board of Jena University Hospital (2910‐08/10, date: 03‐09‐2013) as well as all local ethics committees and works councils responsible for the participating centres. Research was conducted in accordance with the Ethical Principles of the German Psychological Society (DGP) and the Association of German Professional Psychologists (BDP) in their most recent edition. Participation in the survey was completely deliberate. Participants received written information on the aims of the study and the confidentiality of their individual answers. As approved by the ethics committee, no written informed consent was collected, but informed consent was implied if participants returned the completed questionnaire. No written consent has been obtained from the patients as there is no patient‐identifiable data included.

### 3.4 Measures

#### 3.4.1 Staff Questionnaire

The staff questionnaire consisted of demographic items and several published and validated scales. In the following, only the items and scales used for the presented analyses are described. The questionnaire is presented in Supporting Information B.

##### 3.4.1.1 Measures Reported by Nurses

The measurement of psychological safety was based on a 3‐item scale used in a previous study in the context of critical care [16]. To increase reliability we extended this scale by two further items adapted from the original scale on psychological safety developed by Edmondson [17]. Affective organisational commitment to one's ICU was assessed by four items [18]. Commitment to change was assessed by three items [19]. The same 7‐point Likert scale was used for all items (1 = *not at all true*, 7 = *totally true*). Job satisfaction was measured by a single item previously used in the context of critical care indicating satisfaction by one of seven pictures of a face (1 = *strongly angry face*, 7 = *happy smiling face*) [20].

##### 3.4.1.2 Measures Reported by Physicians

We measured the quality of nurse–physician relations by the 3‐item scale on collegial nurse–physician relations from the Nursing Work Index [21], which we extended by adding three additional items from a short scale on interdisciplinary collaboration of teams in neonatal intensive care [22]. To assess shared decision‐making, we used the ‘Collaboration about Care‐Decisions’ scale, which was shortened to four items [23, 24].

#### 3.4.2 Organisational Questionnaire

The organisational questionnaire was developed based on previous literature on constructs and measures relevant to assess structural and organisational characteristics of ICUs or hospital units in general [25, 26, 27, 28]. The organisational questionnaire assessed structural characteristics of the hospital (university hospital, level of care, type of hospital operator) and the ICU (type of ICU, number of beds, number of nurses, number of physicians). The number of nurses was used as a measure of team size. The frequency of participation of the treating nurse in patient rounds was assessed by a single item (5‐point rating scale from 1 = *never* to 5 = *always*). Items to measure nurse autonomy assessed the degree by which an adequately experienced nurse could initiate or regulate typical treatments autonomously on a 4‐point scale (1 = *Nurse determines the necessity of the treatment and initiates it autonomously* to 4 = *Adjustment of therapy is only done after concrete order of physician or by the physician*). The questionnaire is presented in Supporting Information C.

### 3.5 Statistical Analysis

Standard descriptive statistics were used to present the characteristics of hospitals, ICUs and participating staff. Confirmatory factor analyses were used to assess the factorial validity of the measures obtained by the survey of nurses and physicians. Fit was assessed by the root mean square error of approximation (RMSEA < 0.08 for acceptable fit), the comparative fit index (CFI) and the Tucker–Lewis index (TLI; both > 0.9 for acceptable fit). Reliability of scales was assessed by Cronbach's alpha. Scale‐scores were calculated as the mean of the respective items, as long as at least 66% of the items were non‐missing. Since the scales *collegial nurse–physician relations* and *collaboration about care‐decisions* were relatively highly correlated (*r* = 0.67), the mean of both scales was used to measure the quality of nurse–physician relations. As measures of interrater reliability, which is a pre‐requisite to aggregate the individual ratings to the unit level, intraclass correlation coefficient 1—ICC(1)—and ICC(2) were calculated for the scale‐scores [29, 30]. A score of nurses' autonomy per unit was calculated as the mean of the individual items. To give each item roughly equal weight, the item values were first z‐standardised. For investigation of the effects of unit‐level predictors, psychological safety as well as affective commitment to the unit, job satisfaction and commitment to change were aggregated to the unit level by calculating the mean per unit.

To test the effect of unit‐level predictors (number of nurses, nurses' autonomy in patient care, and quality of nurse–physician relations) on nurses' psychological safety, simple linear regression analyses were calculated. To assess mediation, the effects of each predictor on each dependent variable (effect c), the effect of each predictor on the mediator (effect a) and the effects of the mediator on the dependent variables, while controlling for the predictor (effect b) were calculated in linear regression analyses. Significance of the mediation was tested by testing the indirect effect a*b (product of effect a and effect b) against zero by obtaining its 95% confidence interval (CI) using the distribution of the products method [31]. This was done to test the mediation of unit size through quality of nurse–physician relations on psychological safety, as well as to test the mediation of each unit‐level predictor on the endpoints (affective commitment, job satisfaction, commitment to change) through psychological safety. Given the small number of units (*N* = 22), each predictor was assessed separately. Missing data were handled by pairwise deletion; all tests were conducted at a significance level α ≤ 0.05, and analyses were conducted using the statistical software R [32].

## 4 Results

### 4.1 Respondent Characteristics

Characteristics of *N* = 19 participating hospitals and *N* = 22 participating ICUs are presented in Table S1. About half of the hospitals offered tertiary level care (47%); one third were university hospitals (32%); the majority was in public ownership (63%). Participating ICUs had in median 12 beds; nine were mixed ICUs, seven surgical ICUs, five medical ICUs and one was a neurological ICU. In median, 38 nurses were working on the units, of which in median 51% participated in the survey. Of 1216 eligible nurses, 600 (49%) participated (Figure S1). Their characteristics are given in Table 1. The majority was female (75%) and had more than 10 years of experience working as a nurse (61%). About half of the nurses had a certified critical care specialisation (46%). Of 379 eligible physicians, 219 (58%) participated. Their characteristics are presented in Table S2. About two thirds were ward physicians (residents or physicians with board certification), and one third were senior ICU physicians (‘Oberärzte’).

**TABLE 1:** Characteristics of participating nurses.

Variables	*N* (non‐missing)	Descriptive statistics
Age	569	
< 30		178 (31.3%)
30–39		176 (30.9%)
40–49		157 (27.6%)
≥ 50		58 (10.2%)
Sex: female	571	426 (74.6%)
Experience as a nurse (years)	549	
< 1		14 (2.6%)
1–2		36 (6.6%)
3–5		74 (13.5%)
6–10		89 (16.2%)
> 10		336 (61.2%)
ICU experience (years)	545	
< 1		20 (3.7%)
1–2		54 (9.9%)
3–5		103 (18.9%)
6–10		98 (18%)
> 10		270 (49.5%)
Critical care specialisation	535	244 (45.6%)
Working full time on the ICU	534	518 (97%)
Working hours per week	544	40 [32, 40]
Working nights per month	553	5 [4, 6]
Weekend workdays per month	555	5 [4, 5]

*Note:* Descriptive statistics for *N* = 600 participating nurses, given as *N* (%) or median [1st quartile, 3rd quartile].

Abbreviation: ICU: intensive care unit.

### 4.2 Description of Relevant Measures

Tables S3 and S4 present the individual items of the nurse survey and the physician survey, respectively, with their minimum, maximum and mean ratings. Confirmatory factor analyses showed acceptable fit both in the survey of nurses (RMSEA I: 0.076; CFI: 0.945; TLI: 0.928; Table S5) and in the survey of physicians (RMSEA: 0.06; CFI: 0.981; TLI: 0.975; Table S6). Distribution of scale scores is given in Table S7. Reliability as measured by Cronbach's alpha was satisfactory for all scales (α > 0.7, Table S7). ICC1 and ICC2 values indicated that the individual ratings could reliably be aggregated to the unit level (Table S7). Psychological safety showed an ICC1 of 0.21, which means that 21% of the variance in individual ratings of nurses can be attributed to differences in psychological safety between ICUs.

Individual items on nurses' autonomy in patient care are presented in Tables 2 and 3. While some treatments were conducted autonomously by nurses on most units (e.g., changing dressings of central venous catheter or respiratory tube), the autonomy of nurses varied strongly across units for most treatments.

**TABLE 2:** Nurses' autonomy to conduct specific treatments as rated by the leading intensivist of the intensive care unit.

	No answer/treatment not conducted on unit	Nurse determines necessity of the treatment and conducts it autonomously	Nurse conducts treatment autonomously after physician's prescription	Treatment is only conducted by physician or under physician's direct control
Laboratory testing (blood gas analysis)	0	13 (54.2%)	11 (45.8%)	0 (0%)
Insertion of new indwelling venous cannula	0	7 (29.2%)	13 (54.2%)	4 (16.7%)
Insertion of new indwelling bladder catheter	0	16 (66.7%)	8 (33.3%)	0 (0%)
Changing dressings of central venous catheter	0	21 (87.5%)	3 (12.5%)	0 (0%)
Changing dressings of respiration tube	0	21 (87.5%)	3 (12.5%)	0 (0%)
Conduction of weaning from ventilation	1	2 (8.7%)	14 (60.9%)	7 (30.4%)
Moving to prone position	1	1 (4.3%)	8 (34.8%)	14 (60.9%)

*Note:* Descriptive statistics given as *N* (%). Results of survey with the organisational questionnaire of the leading intensivist(s) of the 22 participating intensive care units (ICUs). *N* = 24 intensivists participated since two ICUs were led by two separate departments (anaesthesiology and internal medicine) but shared the same nursing staff. If the same ICU was judged by more than one intensivist, the sum‐score of items of the intensivists was averaged for the further analyses. The instruction for the items was: ‘Below you find a list of therapeutic procedures. Please judge the degree by which nurses on your ICU conduct these procedures autonomously. Make your judgement considering what is usual the case for adequately experienced nurses on your unit’.

**TABLE 3:** Nurses' autonomy to begin or to adjust specific treatments as rated by the leading intensivist of the intensive care unit.

	No answer/treatment not conducted on unit	Nurse determines the necessity of the treatment and initiates it autonomously	Nurse determines necessity of adjustment of treatment and conducts it autonomously	Physician sets concrete target values for parameters and nurse adjust the therapy to achieve the target values	Adjustment of therapy is only done after concrete order of physician or by the physician
Adjustment of oxygen concentration during ventilation	0	—	10 (41.7%)	9 (37.5%)	5 (20.8%)
Adjustment of peak pressure and breathing rate during ventilation	0	—	2 (8.3%)	9 (37.5%)	13 (54.2%)
Adjustment of positive end‐expiratory pressure during ventilation	0	—	2 (8.3%)	9 (37.5%)	13 (54.2%)
Adjustment of calcium and citrate during dialysis	2	—	16 (72.7%)	5 (22.7%)	1 (4.5%)
Catecholamine therapy	0	3 (12.5%)	7 (29.2%)	14 (58.3%)	0 (0%)
Control of sedation	0	2 (8.3%)	6 (25%)	13 (54.2%)	3 (12.5%)
Control of blood glucose through administration of insulin	0	6 (25%)	9 (37.5%)	9 (37.5%)	0 (0%)
Administration of analgesics	0	4 (16.7%)	6 (25%)	11 (45.8%)	3 (12.5%)
Intravenous administration of antihypertensive agents	0	1 (4.2%)	1 (4.2%)	12 (50%)	10 (41.7%)
Administration of crystalloid infusions	0	3 (12.5%)	3 (12.5%)	11 (45.8%)	7 (29.2%)
Administration of caogulation factors	0	0 (0%)	0 (0%)	4 (16.7%)	20 (83.3%)
Control of potassium at the syringe pump	0	4 (16.7%)	7 (29.2%)	11 (45.8%)	2 (8.3%)
Administration of oxygen via nasal tube or mask	0	9 (37.5%)	11 (45.8%)	4 (16.7%)	0 (0%)

*Note:* Descriptive statistics given as *N* (%). Results of survey with the organisational questionnaire of the leading intensivist(s) of the 22 participating intensive care units (ICUs). *N* = 24 intensivists participated since two ICUs were led by two separate departments (anaesthesiology and internal medicine) but shared the same nursing staff. If the same ICU was judged by more than one intensivist, the sum‐score of items of the intensivists was averaged for the further analyses. The instruction for the items was: ‘Below you find a list of therapeutic procedures. Please judge the degree by which nurses on your ICU conduct these procedures autonomously. Make your judgement considering what is usual the case for adequately experienced nurses on your unit’.

### 4.3 Prediction of Psychological Safety

Table S8 presents descriptive statistics and correlations between the study measures aggregated to the unit level. Table 4 gives the effects of simple linear regressions to predict psychological safety (effect a). All predictors (number of nurses working on unit, nurses' always participating in rounds, nurses' autonomy in patient care and quality of nurse–physician relations) showed the expected statistically significant effects on psychological safety, thereby confirming Hypotheses 1 to 4a. The effect of team size was mediated by the quality of nurse–physician relations, since the 95% CI of the indirect effect did not contain zero (indirect effect a*b of −0.19 [95% CI: −0.41, −0.004]). This confirms Hypothesis 4b (effect of team size partly mediated by quality of nurse–physician relations).

**TABLE 4:** Tests of hypotheses.

Predictor	Effects (a) of predictors on psychological safety (mediator)		Effect (b) of psychological safety on endpoints		Direct effect (c′) of predictors on endpoints		Indirect effect of predictors on endpoints mediated by psychological safety (a*b)	Total effect of predictor on endpoint (c)	
	Effect (95% CI)	*p*	Effect (95% CI)	*p*	Effect (95% CI)	*p*	Effect (95% CI)	Effect (95% CI)	*p*
Endpoint job satisfaction									
Number of nurses working on unit^a^	−0.55 (−0.94, −0.16)	0.008	0.71 (0.41, 1)	≤ 0.001	−0.23 (−0.53, 0.06)	0.117	−0.37 (−0.6, −0.15)	−0.62 (−0.99, −0.25)	0.002
Nurses always participating in rounds^a^	0.51 (0.11, 0.91)	0.014	0.73 (0.44, 1.02)	≤ 0.001	0.2 (−0.09, 0.49)	0.167	0.36 (0.12, 0.61)	0.58 (0.2, 0.96)	0.005
Nurses' autonomy in patient care^a^	0.51 (0.1, 0.91)	0.016	0.68 (0.41, 0.95)	≤ 0.001	0.31 (0.04, 0.58)	0.025	0.33 (0.1, 0.56)	0.65 (0.3, 1.01)	≤ 0.001
Quality of nurse–physician relations^b^	0.59 (0.21, 0.97)	0.004	0.75 (0.43, 1.07)	≤ 0.001	0.15 (−0.17, 0.47)	0.337	0.42 (0.2, 0.65)	0.59 (0.21, 0.97)	0.004
Endpoint affective commitment to the unit									
Number of nurses working on unit^a^	−0.55 (−0.94, −0.16)	0.008	0.71 (0.41, 1)	≤ 0.001	−0.23 (−0.53, 0.06)	0.117	−0.37 (−0.6, −0.15)	−0.62 (−0.99, −0.25)	0.002
Nurses always participating in rounds^a^	0.51 (0.11, 0.91)	0.014	0.73 (0.44, 1.02)	≤ 0.001	0.2 (−0.09, 0.49)	0.167	0.36 (0.12, 0.61)	0.58 (0.2, 0.96)	0.005
Nurses' autonomy in patient care^a^	0.51 (0.1, 0.91)	0.016	0.68 (0.41, 0.95)	≤ 0.001	0.31 (0.04, 0.58)	0.025	0.33 (0.1, 0.56)	0.65 (0.3, 1.01)	≤ 0.001
Quality of nurse–physician relations^b^	0.59 (0.21, 0.97)	0.004	0.75 (0.43, 1.07)	≤ 0.001	0.15 (−0.17, 0.47)	0.337	0.42 (0.2, 0.65)	0.59 (0.21, 0.97)	0.004
Endpoint commitment to change									
Number of nurses working on unit^a^	−0.55 (−0.94, −0.16)	0.008	0.71 (0.41, 1)	≤ 0.001	−0.23 (−0.53, 0.06)	0.117	−0.37 (−0.6, −0.15)	−0.62 (−0.99, −0.25)	0.002
Nurses always participating in rounds^a^	0.51 (0.11, 0.91)	0.014	0.73 (0.44, 1.02)	≤ 0.001	0.2 (−0.09, 0.49)	0.167	0.36 (0.12, 0.61)	0.58 (0.2, 0.96)	0.005
Nurses' autonomy in patient care^a^	0.51 (0.1, 0.91)	0.016	0.68 (0.41, 0.95)	≤ 0.001	0.31 (0.04, 0.58)	0.025	0.33 (0.1, 0.56)	0.65 (0.3, 1.01)	≤ 0.001
Quality of nurse–physician relations^b^	0.59 (0.21, 0.97)	0.004	0.75 (0.43, 1.07)	≤ 0.001	0.15 (−0.17, 0.47)	0.337	0.42 (0.2, 0.65)	0.59 (0.21, 0.97)	0.004

*Note:* Effects obtained by linear regressions at the unit level. Variables measured at individual level were aggregated by taking the mean per unit. Significance of the mediation was tested by testing the indirect effect (a*b) against zero by obtaining its 95% confidence interval (CI) using the distribution of the products method [31]; if the CI does not contain zero, the mediation effect through psychological safety is statistically significant.

^a^ Measure obtained by survey of leading intensivists of the ICU.

^b^ Measure obtained by survey of ICU physicians.

### 4.4 Psychological Safety as a Mediator

Table 4 represents the results of the mediation analyses for psychological safety. All predictor variables (number of nurses working on unit, nurses' always participating in rounds, nurses' autonomy in patient care and quality of nurse–physician relations) showed the expected statistically significant effects on job satisfaction, affective commitment to the unit and commitment to change (effects c in Table 4). Since the 95% CI of the indirect effects (a*b) did not contain zero, the mediation effects by psychological safety were all statistically significant, as was hypothesised. This confirms Hypotheses 5.1a to 5.4c.

## 5 Discussion

### 5.1 Summary of Findings

This study found that smaller team size, greater nurse autonomy, consistent nurse participation in ward rounds and higher quality nurse–physician collaboration were each associated with greater psychological safety among ICU nurses in Germany These predictors were also associated with job satisfaction, affective commitment to the unit and commitment to change, while these associations were mediated by psychological safety.

### 5.2 Interpretation and Practical Implications

To our knowledge, no study has quantitatively assessed the association between ICU nurse team size and psychological safety, nor has this association been quantitatively assessed in any healthcare setting or such a large sample before. It has been strongly argued that larger ICUs are more cost‐effective [33, 34, 35] and might result in better outcomes [36, 37]. Therefore, there is a trend towards larger intensive care units. There is also a tendency to deploy critical care nurses to different units within a hospital for better personnel flexibility and to fill roster gaps by travel nurses, all resulting in larger numbers of nurses working in a unit and potentially impeding the development of durable good nurse–physician relations. For surgeon–anesthesiologist dyads and critical care nursing shifts, it was recently shown that a higher familiarity, measured by the number of cases or shifts worked together, was associated with better outcomes [38, 39]. Psychological safety could be a factor contributing to this association.

Interprofessional ward rounds have previously been shown to influence patient care and teamwork [40] and their influence on psychological safety shown by us fits well with those previous findings. The association of nurse–physician relations on psychological safety has previously only been demonstrated in qualitative research [41]. Our findings suggest that smaller teams with good nurse–physician relations and regular interprofessional communication are associated with psychological safety. Interestingly, there are publications showing an association of measured nursing team familiarity with patient outcome in the ICU [39, 42] and higher psychological safety could partly explain these findings. Therefore, there might be a relevant trade‐off between economics of scale and psychological safety of nurses in critical care or even healthcare in general that might even affect patient outcome [43].

Perceived autonomy in patient care is of great importance for nurses' job satisfaction [44, 45, 46] and its association with psychological safety has been demonstrated in mental health nurses [47]. The ICU, where many specific tasks can be performed by physicians or nurses, is a good setting to measure autonomy in an objective, reproducible way. In our study, autonomy in the intensive care unit was assessed as objectively as possible by asking the units' leading intensivists for nurse autonomy in a range of different, specific tasks within the scope of critical care nursing. We observed high heterogeneity regarding overall autonomy and responsibility for specific tasks even within one legal framework. Our results are in line with a finding from a national survey of German critical care units conducted in 2017. In 78% of participating ICUs, specialised critical care nurses did not have higher qualified work tasks in comparison with general nurses working in the same unit; only 1% of units reported relevant differences [48]. There is limited international data on heterogeneities in ICU nurse autonomy across institutions or healthcare systems [49, 50]. There might be an unused potential to increase psychological safety by enabling higher autonomy for highly qualified nurses, but more data are needed.

Global critical care nurse shortages, exacerbated by the COVID‐19 pandemic, underscore the need to enhance wellbeing and retainment of highly qualified staff [51, 52]. Our findings about the effects of team size, nurse–physician relations, interprofessional rounds and autonomy on job satisfaction and emotional commitment mediated by psychological safety give actionable insights how to forward this goal. Despite the requirement for continual change and development, change failure is omnipresent in healthcare [53] and increasing psychological safety and consecutively commitment to change is therefore another desirable goal.

### 5.3 Strengths and Limitations

The questionnaire survey is one of the major methods to assess the work environment of nurses but can be biased by self‐report and common method bias [54, 55]. Therefore, the major strength of our study is its multi‐informant method, which assessed endpoints by a survey of nurses, but influencing factors by an organisational questionnaire and survey of physicians. A second strength is the assessment of relations on the unit level, which prevents an atomistic fallacy by drawing conclusions from the individual survey data to the level of teams or units [56]. The third strength is the thorough development of a new instrument to assess nurses' autonomy in patient care using highly specific tasks traditionally conducted by physicians. Whereas previous studies on nurses' autonomy used general and subjective rating scales, this approach allowed for a more objective and independent assessment of specific intensive care tasks. A further strength is the structured development of the staff survey and extensive validation by confirmatory factor analysis and assessment of interrater reliability. A limitation is the limited number of participating units, which prevented us from assessing the different unit‐level variables in a common model. Our data are 10 years old. Anyhow, we believe that the multicausal delay of publishing does not cause a relevant lack of continued relevancy of our findings. First, we focused on assessing relationships between variables, which are less prone to secular changes compared to rates or means. Second, no relevant changes of the classical hierarchical relations between physicians and nurses have occurred during the last decade in the German healthcare system. We investigated aspects of nurse–physician collaboration in the context of critical care, which might not be fully generalisable to other disciplines. It is also important to point out that statistical effects found in our dataset do not prove causality.

## 6 Conclusion

This study confirms the important role of psychological safety as a core characteristic of successful healthcare teams, since it predicts essential outcomes of critical care nurses' job well‐being. In addition, we found that psychological safety can be enhanced by appropriately designing aspects of the interprofessional collaboration on the ICU. Critical care departments should strive for changing their organisation to smaller, stable care teams, which allow better development of trusting interpersonal relations. Physicians, especially leading intensivists and attendings, play a major role for the psychological safety of nurses in the interprofessional team. They should invite nurses' contributions and shared decision‐making on patient care, and they should empower nurses to take greater responsibilities classically associated with the physician's role. Future studies should assess the applicability of our findings to other medical disciplines, especially in the setting of the normal ward and to other countries with different legal and cultural frameworks.

## 7 Implication for Clinical Practice

Fostering interprofessional collaboration and enhancing nurses' autonomy in critical care are key to enhancing psychological safety and thereby enhancing job satisfaction and retention. Increasing team size can have unintended negative consequences that need to be weighted against potential gains.

## Author Contributions

**D.O.T.‐R.:** conceptualization, investigation, writing – original draft, writing – review and editing, project administration. **F.B.:** investigation, resources, writing – review and editing, supervision, project administration, funding acquisition. **H.R.:** investigation, writing – review and editing, project administration. **D.S.:** conceptualization, methodology, software, formal analysis, investigation, data curation, writing – review and editing, visualization, project administration.

## Funding

This work was supported by the German Federal Ministry of Education and Research via the integrated research and treatment centre ‘Center for Sepsis Control and Care’ (FKZ 01EO1002). The funding organisation had no influence on the survey's design, implementation or analysis.

## Ethics Statement

The study was approved by the institutional review board of Jena University Hospital (2910‐08/10, date: 03‐09‐2013) as well as all local ethics committees and works councils responsible for the participating cenres. Research was conducted in accordance with the Ethical Principles of the German Psychological Society (DGP) and the Association of German Professional Psychologists (BDP) in their most recent edition. Participation in the survey was completely deliberate. Participants received written information on the aims of the study and the confidentiality of their individual answers. As approved by the ethics committee, no written informed consent was collected, but informed consent was implied if participants returned the completed questionnaire. No written consent has been obtained from the patients as there is no patient‐identifiable data included.

## Consent

The authors have nothing to report.

## Conflicts of Interest

The authors declare no conflicts of interest.

## Supporting information


**Supporting Information: A** A translation of the used staff questionnaire.

**Supporting Information: B** An extended description of the development of the research hypotheses and the development of the staff questionnaire.

**Supporting Information: C.** A translation of the centre level questionnaire.

**Supporting Information D. Supplemental Figures and Tables. Figure S1:** Study flow chart. Response rate defined as number of returned questionnaires divided by number of staff reported by local study coordinators.
**Table S1:** Characteristics of participating hospitals and intensive care units.
**Table S2:** Characteristics of participating physicians.
**Table S3:** Items of the survey of nurses.
**Table S4:** Items of the survey of physicians.
**Table S5:** Factor loadings of confirmatory factor analysis of items of survey of nurses.
**Table S6:** Factor loadings of confirmatory factor analysis of items of survey of physicians.
**Table S7:** Distribution of scale scores, internal consistency and interrater reliability.
**Table S8:** Descriptive statistics and correlations for study measures aggregated to the unit level.

## Data Availability

Research data cannot be shared due to German and European data protection regulations.
