# Supplementary material for: Designing Nurse–Physician Collaboration to Improve Psychological Safety, Satisfaction and Commitment of Critical Care Nurses—A Multi‐Informant Survey Study
Source: Nurs Crit Care. 2026 Jul 1;31(4):e70567. doi: 10.1111/nicc.70567 (PMC13320614; doi:10.1111/nicc.70567)
Supplement: Supplementary file 1 — Supporting Information: A A translation of the used staff questionnaire. [file NICC-31-0-s001.docx]

**
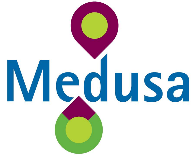
**

**Information Sheet for the Employee Survey of the MEDUSA Study**

Thank you for your willingness to participate in the survey. We would like to briefly inform you about the most important points:

**The protection of the confidentiality of your information is our highest priority!**

- The survey has been approved by your institution’s staff representation and is monitored by the Data Protection Officer of Jena University Hospital.
- **Completed questionnaires are sent directly by post to the MEDUSA study team in the enclosed return envelope.**
- Only members of the MEDUSA study have access to the questionnaires and the collected data.
- Reports of results and any other presentation of the results to third parties are carried out in such a way that **no** conclusions can be drawn about individual respondents (only aggregated data, e.g. mean values, based on a minimum of 10 participating persons).
- Assessments of supervisors in the survey are also presented only in aggregated form if they refer to at least 5 persons.

**Instructions for Completing the Questionnaire**

- Completing the questionnaire takes approximately 15–20 minutes.
- Please complete the questionnaire in full.
- If you wish to leave out individual questions, please still send us the incomplete questionnaire.
- Please always select exactly one alternative from the response scales by ticking the appropriate box.
-
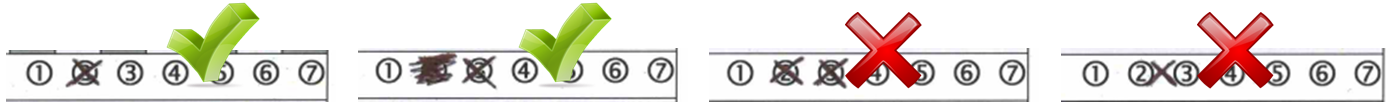
If you wish to change your answer, clearly cross out the previous mark:
- In this questionnaire, “ward physicians” refers to all resident physicians and board certified physicians who are not senior physicians.

**What Are the Benefits of Participation?**

- The results will be compared with those of other wards throughout Germany (“benchmarking”).
- The survey results will be communicated to all employees (detailed report of results, posters, information sheets).
- The results provide an information base for problem analysis and improvements.

**If you have any questions, please contact:**

| **Contact person at your institution:**  Title First Name Last Name  Position  Department  Address (if applicable)  Tel:  E-mail: | **Contact person for MEDUSA:**  Title First Name Last Name Position  Department  Adress:  Tel:  E-mail: |
| --- | --- |

| **1** | **2** | **3** | **4** | **5** | **6** | **7** |
| --- | --- | --- | --- | --- | --- | --- |
| **does not apply at all** | **largely does not apply** | **rather does not apply** | **partly applies / partly does not apply** | **rather applies** | **largely applies** | **fully applies** |

Please rate, using the scale shown above, to what extent the following statements apply to your work situation in your ICU.

|  | | | | | **1** | **2** | | **3** | **4** | | **5** | **6** | | **7** |
| --- | --- | --- | --- | --- | --- | --- | --- | --- | --- | --- | --- | --- | --- | --- |
| I am proud to belong to this unit. | | | | | **➀** | **➁** | | **➂** | **➃** | | **➄** | **➅** | | **➆** |
| I feel a strong sense of belonging to my unit. | | | | | **➀** | **➁** | | **➂** | **➃** | | **➄** | **➅** | | **➆** |
| I think that my moral values fit those of the unit. | | | | | **➀** | **➁** | | **➂** | **➃** | | **➄** | **➅** | | **➆** |
| I **don’t** feel particularly emotionally attached to this unit. | | | | | **➀** | **➁** | | **➂** | **➃** | | **➄** | **➅** | | **➆** |
| Overall, how satisfied are you with your job on your unit? | 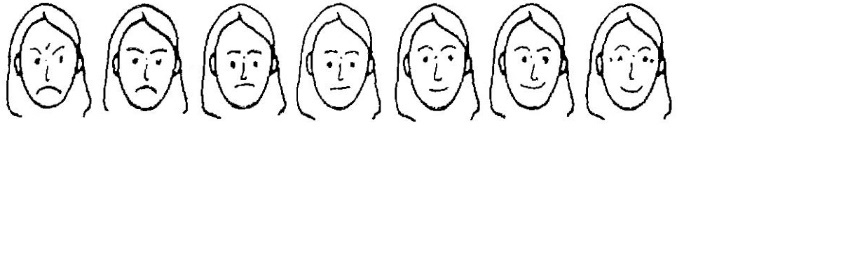 | | | | | | | | | | | | | |
|  | ⭘ | ⭘ | ⭘ | ⭘ | | | ⭘ | | | ⭘ | | | ⭘ | |

| **1** | **2** | **3** | **4** | **5** | **6** | **7** |
| --- | --- | --- | --- | --- | --- | --- |
| **does not apply at all** | **largely does not apply** | **rather does not apply** | **partly applies / partly does not apply** | **rather applies** | **largely applies** | **fully applies** |

Please rate, using the scale shown above, to what extent the following statements apply to your ICU team.

|  | **1** | **2** | **3** | **4** | **5** | **6** | **7** |
| --- | --- | --- | --- | --- | --- | --- | --- |
| Physicians and nurses have good working relationships. | **➀** | **➁** | **➂** | **➃** | **➄** | **➅** | **➆** |
| A lot of team work between nurses and physicians. | **➀** | **➁** | **➂** | **➃** | **➄** | **➅** | **➆** |
| Communication between nurses and physicians on our unit is open and positive. | **➀** | **➁** | **➂** | **➃** | **➄** | **➅** | **➆** |
| When there is a disagreement between nurses and physicians of our unit, all points of view will be carefully considered in arriving at the best solutions to the problem. | **➀** | **➁** | **➂** | **➃** | **➄** | **➅** | **➆** |
| Collaboration (joint patient care) between nurses and physicians is good. | **➀** | **➁** | **➂** | **➃** | **➄** | **➅** | **➆** |
| Nurses and physicians of our unit have a good understanding of each other’s tasks and responsibilities. | **➀** | **➁** | **➂** | **➃** | **➄** | **➅** | **➆** |
| Nurses in this unit are comfortable checking with other nurses or physicians of the team if they have questions about the right way to do something.^[[1]](#footnote-1)^ | **➀** | **➁** | **➂** | **➃** | **➄** | **➅** | **➆** |
| The nurses in this unit are able to bring up problems and tough issues.^1^ | **➀** | **➁** | **➂** | **➃** | **➄** | **➅** | **➆** |
| Nurses who state a differing opinion are often confronted with rejection in this unit.^1^ | **➀** | **➁** | **➂** | **➃** | **➄** | **➅** | **➆** |
| If a nurse makes a mistake in this unit, it is often held against him.^1^ | **➀** | **➁** | **➂** | **➃** | **➄** | **➅** | **➆** |
| For nurses in this unit it is difficult to ask other nurses or physicians of the team for help.^1^ | **➀** | **➁** | **➂** | **➃** | **➄** | **➅** | **➆** |
| Nurses and physicians plan together to make decisions about care for patients. | **➀** | **➁** | **➂** | **➃** | **➄** | **➅** | **➆** |
| Open communication between physicians and nurses takes place as decisions are made for patients. | **➀** | **➁** | **➂** | **➃** | **➄** | **➅** | **➆** |
| Physicians and nurses cooperate in making decisions regarding patient care. | **➀** | **➁** | **➂** | **➃** | **➄** | **➅** | **➆** |
| In making decisions about patient care, both nursing and medical concerns are considered. | **➀** | **➁** | **➂** | **➃** | **➄** | **➅** | **➆** |

| **1** | **2** | **3** | **4** | **5** | **6** | **7** |
| --- | --- | --- | --- | --- | --- | --- |
| **does not apply at all** | **largely does not apply** | **rather does not apply** | **partly applies / partly does not apply** | **rather applies** | **largely applies** | **fully applies** |

To improve patient care, changes in work processes on the unit may be necessary. Please consider how such change processes are implemented in your ICU. Then rate, using the scale shown above, to what extent the following statements apply

|  | **1** | **2** | **3** | **4** | **5** | **6** | **7** |
| --- | --- | --- | --- | --- | --- | --- | --- |
| I believe that we are actively approaching changes in working processes to improve patient care instead of just reacting, if necessary. | **➀** | **➁** | **➂** | **➃** | **➄** | **➅** | **➆** |
| I believe there will be changes of working processes in our unit that will improve the quality of patient care in the next years. | **➀** | **➁** | **➂** | **➃** | **➄** | **➅** | **➆** |
| I think that I have no possibility to influence changes in working processes for the improvement of patient care on this unit. | **➀** | **➁** | **➂** | **➃** | **➄** | **➅** | **➆** |

| Your age (in years)^[[2]](#footnote-2)^:  ⭘ < 30 ⭘ 30 – 39 ⭘ 40– 49 ⭘ ≥ 50  Medically active (since completion of training) (years): ⭘ < 1 ⭘ 1 - 2 ⭘ 3 - 5 ⭘ 6 -10 ⭘ > 10 | | Working in intensive care (since completion of training) (years): ⭘ < 1 ⭘ 1 - 2 ⭘ 3 - 5 ⭘ 6 -10 ⭘ > 10 |
| --- | --- | --- |
| Gender: ⭘ male  ⭘ female | How many hours per week do you work in the hospital on average?: _____  Of these, how many hours are spent in intensive care (hours)?: _____  On average, how many nights do you work per month? (in total, not only in the ICU): _____  On average, how many days per month do you work on weekends? (in total, not only in the ICU): _____ | |
| You are : ⭘ Nurse  ⭘ Nurse manger (unit level) |  |  |
| Specialist ICU and anesthesia nurse  ⭘ yes ⭘ no |  |  |

| Your age (in years):^[[3]](#footnote-3)^  ⭘ < 30 ⭘ 30 – 39 ⭘ 40– 49 ⭘ ≥ 50  Medically active (since graduation) (years): ⭘ < 1 ⭘ 1 - 2 ⭘ 3 - 5 ⭘ 6 -10 ⭘ > 10 | | Working in intensive care (since graduation) (years): ⭘ < 1 ⭘ 1 - 2 ⭘ 3 - 5 ⭘ 6 -10 ⭘ > 10 |
| --- | --- | --- |
| Gender: ⭘ male  ⭘ female | How many hours per week do you work in the hospital on average?: _____  Of these, how many hours are spent in intensive care (hours)?: _____  On average, how many nights do you work per month? (in total, not only in the ICU) On-call duty:_____ in-house:_____  On average, how many days per month do you work on weekends? (in total, not only in the ICU): On-call duty:_____ in-house:_____ | |
| You are: ⭘ Resident  ⭘ board certified physician^[[4]](#footnote-4)^ |  |  |
| Additional qualification in intensive care medicine  ⭘ yes ⭘ no |  |  |

1. Items on the psychological safety of nurses were presented to nurses, while ward physicians rated the psychological safety for ward physicians and senior attendings rated the psychological safety as they perceived it for these two groups. Only the assessment of psychological safety by the nurses themselves were used in the reported analyses. [↑](#footnote-ref-1)
2. Demographic items for the nurse questionnaire. [↑](#footnote-ref-2)
3. Demographic items for the physician questionnaires. [↑](#footnote-ref-3)
4. In Germany there is the additional position of “Oberarzt” which is senior ICU physician supervising residents and fellows, managing care, with significant clinical leadership responsibilities. Senior attendings received a separate version of the questionnaire and were thereby identified. [↑](#footnote-ref-4)
