# Supplementary material for: Designing Nurse–Physician Collaboration to Improve Psychological Safety, Satisfaction and Commitment of Critical Care Nurses—A Multi‐Informant Survey Study
Source: Nurs Crit Care. 2026 Jul 1;31(4):e70567. doi: 10.1111/nicc.70567 (PMC13320614; doi:10.1111/nicc.70567)
Supplement: Supplementary file 2 — Supporting Information: B An extended description of the development of the research hypotheses and the development of the staff questionnaire. [file NICC-31-0-s003.docx]

Supplementary Material A – Supplementary Text

# Development of hypotheses

## Nurse’s autonomy in performing ICU patient care

In job characteristics theory job autonomy refers to the degree to which employees can make decisions independently and have autonomy in planning and executing tasks and is considered a key characteristic influencing job motivation and satisfaction(1). This was also specifically shown for intensive care nursing (2). For research and development contexts, where autonomy can involve a very high degree of freedom in planning, designing and managing tasks, a correlation with psychological safety has already been shown (3).

In nursing, the clinical autonomy of staff nurses who provide direct patient care refers to their ability to act beyond standard practice and make decisions regarding individual patients’ care (4). It also involves responsibility for decisions and freedom to make clinical choices (4). As increased autonomy promotes confidence in nurse’s own knowledge, good interprofessional collaboration and responsibility for the care process and decreases the perceived difference in professional status it should result in higher psychological safety.

Hypothesis 1: Autonomy and responsibility of nurses as reported by the leading attending/medical director of an ICU is positively associated with psychological safety.

## Nurses participation in ward rounds

Ward rounds (WRs) are a complex social process. Health care professionals meet at the bedside to review and plan care (5). The German Interdisciplinary Society of Intensive Care Medicine (DIVI) in 2013 defined daily interprofessional ward rounds including at least nurse and physician as a quality indicator in the ICU (6, 7). In a recent survey of German ICUs with 390 respondents only 51% reported that ICU nurses are always present during rounds, 36% reported them to be usually present (8). Studies of nurse-consultant interactions and collaboration during WRs suggest that if nurses perceive that their views are not being heard, or they view WRs as ritualistic, they may disengage from interaction whilst remaining physically present, or even absent themselves (5). Leader inclusiveness is when leaders demonstrate availability, openness, and accessibility to team members by inviting input and offering appreciation for their employees’ contributions (9). Interprofessional WRs are one of the most important opportunities for senior doctors to demonstrate leader inclusiveness and one would assume that leader inclusiveness is associated with a high presence of nurses in WRs. The association of leader inclusiveness with psychological safety is well demonstrated (9-11).

Hypothesis 2: Nurse working in an ICU where nurses always participate in ward rounds feel more psychologically safe.

## Quality of nurse-physician relations

Between nurses and physicians traditionally has been a gap in hierarchical status and power (12, 13) and conflicts and adversarial behaviors are not uncommon despite improvements over time (13, 14). Intensive care units on average report better nurse-physician relationships than normal wards (14). Given the importance of interprofessional cooperation in the ICU this finding is not surprising and the quality of collaboration has even been linked with outcome in critically ill patients (15). As hierarchical differences are a main barrier to psychological safety one would assume that good, collaborative relations foster mutual respect and shared goals and knowledge resulting in increased psychological safety, especially for team members perceived to have lower hierarchical status. This has already been shown for non-healthcare settings (16).

Hypothesis 3: Quality of nurse-physician relations and collaboration as reported by physicians is positively associated with psychological safety in nurses.

## Team size

There is conflicting data on the effect of team size on performance. While some researchers found no influence of team size on performance (17, 18), Edmondson described greatly improved performance after splitting up a large emergency room shift team into smaller teams (19). While the core team caring for a single ICU patient on a given moment is quite small and mostly consists of a nurse, a junior physician and a senior physician, the interprofessional ICU team present in the unit is much bigger. The whole number of nurses working in a given ICU is even bigger and not comparable to the team sizes studied in the mentioned research. The effect of team size on psychological safety was studied for small management or research teams where a negative correlation was observed (3, 20). Team familiarity, which is probably easier to achieve in smaller groups, has been found to be an enabling factor for psychological safety in healthcare teams, but mostly by qualitative research (11, 21, 22). Dunbar’s Number (23), the theory about a precisely fixed upper level of steady social relations a human being can process, has been mostly dismissed (24, 25). However, with increasing numbers of group members, social complexity increases, especially when there is additional role distinction (26). As a member of a group, it might be easier to calculate the “risk” of speaking up in a small group of well-known individuals (21) with which close social relations exist then in a large group of less well-known individuals with looser personal relations (27).

Hypothesis 4a: Nurses working in an ICU with a smaller number of nurses working in the unit feel more psychologically safe than nurses working in an ICU with a large number of nurses.

Hypothesis 4b: The effect of team size on psychological safety is partly mediated by the quality of nurse-physician relations.

## Psychological safety as mediator to job satisfaction and organizational commitment

Affective commitment is a form of organizational commitment that refers to employees’ emotional dependence on, identification with, and involvement in an organization. This inclination to maintain long-term affiliation with the organization fosters a heightened emotional bond with the institution (28). Job satisfaction is defined as an employee’s cognitive and emotional evaluation of their job, which has either a positive or negative form (29). Both are associated with desired organizational outcomes, especially increased job performance and reduced staff turnover (30, 31). Positive nurse-physician collaboration has been shown to predict both job satisfaction and commitment of nurses (32). In addition, affective commitment and job satisfaction have been proposed as an outcome of psychological safety (33) and their association in the health care sector has also been shown before (34). The constant need to implement medical and technical innovations in health care organizations also puts commitment to change in focus, which is the major prerequisite for successful change processes (35). One major obstacle to organizational learning in hospitals are hierarchical inter-professional interactions which result in failures of health care teams to learn collaboratively (36). On the other hand, psychological safety is known to be fostered by high quality work relations and to enhance learning in work teams (16). We therefore assume that the different aspects of a positive nurse-physician collaboration are associated with the organizational outcomes of increased affective commitment, increased job satisfaction, and increased commitment to change of nurses, and that those associations are mediated by psychological safety.

Hypothesis 5.1: psychological safety mediates a positive effect of nurses’autonomy in patient care on (a) job satisfaction, (b) affective commitment to the unit, and (c) commitment to change.

Hypothesis 5.2: psychological safety mediates a positive effect of nurses’ participation in WR on (a) job satisfaction, (b) affective commitment to the unit, and (c) commitment to change.

Hypothesis 5.3: psychological safety mediates a positive effect of the quality of nurse-phyisician relations on (a) job satisfaction, (b) affective commitment to the unit, and (c) commitment to change.

Hypothesis 5.4: psychological safety mediates the negative effect of unit size on (a) job satisfaction, (b) affective commitment to the unit, and (c) commitment to change.

# Supplementary methods

## Details on design and setting

Hospitals involved in an ongoing multicenter cluster-randomized trial to improve treatment of severe sepsis (Medical Education for Sepsis Source Control and Antibiotics, MEDUSA, ClinicalTrials.gov Identifier NCT01187134) (37, 38) were invited to participate in the survey in autumn 2013. Sixteen of 36 invited hospitals participated. Three more hospitals not involved in the MEDUSA trial group participated because of personal interest. Three centers participated with two intensive care units (ICUs) each; one center participated with three ICUs. In one center, two ICUs were combined to one since they shared one common nursing team. This resulted in 22 ICUs for analysis. Despite the use of a convenience sample, the participating hospitals represent the diversity of intensive care services in Germany in terms of care levels and regional distribution. No a-priory sample size calculation was conducted. This study was conducted based on funding for the MEDUSA trial provided by the German Federal Ministry of Education and Research via the integrated research and treatment center “Center for Sepsis Control and Care” (FKZ 01EO1002). The funder hat no role in the design, implementation, or analysis of the survey.

## Details on procedure

The survey was distributed in three versions differentiating three roles on the ICU: nurses, ward physicians (“Stationsärzte”), senior attendings (“Oberärzte”)^[[1]](#footnote-1)^. This was done to prevent missing data on the job role and because some items were used in specific wording regarding the job role (see Supplemental Material B). To increase the response rate, each local investigator received feedback on the rate of participation per role within his hospital after two and four weeks, and questionnaires were distributed a second time after four weeks.

Demographic information was collected only in broad categories to ensure the greatest possible anonymity of respondents; individual survey results were treated with strict confidentiality by the study team. Data were entered into a Microsoft Access 2010 database (Microsoft Corporation, Redmond, WA) by student assistants using structured data entry forms to minimize input errors and ensure consistency.

## Details on the development of the staff questionnaire

Items not available in German were translated from English by a forward and backward translation process. Items were discussed in the research team and with experienced critical care nurses and physicians to assess their fit to the context of critical care. If necessary items not fitting to the context were adapted or discarded, if proper adaptation was not possible. To establish face validity, comprehensiveness and feasibility of the resulting item set, it was pre-tested by cognitive interviews among 17 ICU nurses and physicians. Final changes to item wordings were done, if necessary. A quantitative pre-test was done in a pilot phase in two of the 19 study hospitals involving 220 nurses and 55 physicians. Based on analyses of item characteristics and exploratory factor analyses, some items were excluded from the questionnaire before the survey was conducted in all other centers. All data from the pilot phase was included in the reported analyses. The changes done to the validated scales during the development process are described in the following. The final item are presented in Supplementary Material D, Table D.3 and in Supplemental Material B.

Psychological safety: This construct was assessed by three items in a study among ICU staff (39). These items had been adapted from a previous scale developed for work teams in manufacturing by Edmondson (40). To increase reliability we selected three further items from this original scale (see below). Two items, which did not seem fitting for the ICU context (“It is safe to take a risk on this team”, “No one on this team would deliberately act in a way that undermines my efforts.”), were disregarded. All items were adapted in wording to refer to nurses. After the pilot phase, one item was disregarded, since it did not load on the same factor as the other items. This resulted in the five items used to assess psychological safety in the main study (see Table A. 1.).

**Table A.1. Development of the psychological safety scale**

| **Original item** | **Adapted item** | **Included in final survey** |
| --- | --- | --- |
| People in this unit are comfortable checking with each other if they have questions about the right way to do something. (39) | Nurses in this unit are comfortable checking with other nurses or physicians of the team if they have questions about the right way to do something. | yes |
| The people in our unit value others’ unique skills and talents. (39) | In our unit the unique skills and knowings of the individual nurses are valued. | no^a^ |
| Members of this NICU are able to bring up problems and tough issues. (39) | The nurses in this unit are able to bring up problems and tough issues. | yes |
| People on this team sometimes reject others for being different. (40) | Nurses who state a differing opinion are often confronted with rejection in this unit. | yes |
| If you make a mistake on this team, it is often held against you. (40) | If a nurse makes a mistake in this unit, it is often held against him. | yes |
| It is difficult to ask other members of this team for help. (40) | For nurses in this unit it is difficult to ask other nurses or physicians of the team for help. | yes |

^a^ Excluded based on results of exploratory factor analysis in the pilot phase.

Affective organizational commitment: To measure this concept we used a subscale of a validated instrument already existing in German (41). The original scale had five items. We decided to disregard one item (“I would be very happy to spend the rest of my working life in this organization.”), because it had a strong conceptual overlap with another distinct construct – intention to leave the job, which was assessed in the same survey for our previously reported substudy (42). We adapted the items by exchanging “organization” with “unit” (see Table A.2.).

**Table A.2. Development of the affective organizational commitment scale**

| **Original item** | **Adapted item** | **Included in final survey** |
| --- | --- | --- |
| I am proud to belong to this unit. | I am proud to belong to this unit. | yes |
| I feel a strong sense of belonging to my organization | I feel a strong sense of belonging to my unit. | yes |
| I think that my moral values fit those of the organization. | I think that my moral values fit those of the unit. | yes |
| I don’t feel particularly emotionally attached to this organization. | I don’t feel particularly attached to this unit. | yes |
| I would be very happy to spend the rest of my working life in this organization | - | no^a^ |

^a^ Excluded due to conceptual overlap with the concept of “intention to leave the job”.

Commitment to change: We based the assessment on a scale already available in German (43). This scale referred to change processes in the work context in general. We adapted the item wording to focus on changes in processes to improve patient care. Discussions with experts and cognitive pre-testing led to further adaptations of item wordings, which aimed to retain core concepts of items, but also to fit the context and values of ICU staff. The original scale consisted of six items, but we were unable to replicate the one-factorial structure in the pilot phase. We needed to exclude three items, wich resulted in a final set of three items, which loaded on a common factor (see Table A.3.).

**Table A.3. Development of the commitment to change scale**

| **Original item** | **Adapted item** | **Included in final survey** |
| --- | --- | --- |
| I increasingly feel that the process of change is “my thing.” | Changes of working processes to improve patient care are personally important to me. | no^a^ |
| I have the impression that we will be able to act rather than just react in the future as well. | I believe that we are actively approaching changes in working processes to improve patient care instead of just reacting, if necessary. | yes |
| The process of change in my working environment also benefits me personally | I personally benefit of such changes to working procceses. | no^a^ |
| I am looking forward to what will change to improve the quality of patient care on our unit during the next years | I believe there will be changes of working processes in our unit that will improve the quality of patient care in the next years. | yes |
| I am fundamentally suspicious of all changes. | I generally mistrust changes to working processes for improvement of patient care on our unit. | no^a^ |
| I think that I have no possibility to influence changes. | I think that I have no possibility to influence changes in working processes for the improvement of patient care on this unit. | yes |

^a^ Excluded based on results of exploratory factor analysis in the pilot phase.

Collegial nurse-physician relations: We used the three-item scale on collegial nurse-physician relations from the Nursing Work Index (44), which we extended by adding three additional items from a short scale on interdisciplinary collaboration of teams in neonatal intensive care (45). We adapted the latter items to refer to the relation between nurses and physicians (see Table A.4.). Only minor further adaptations of the items text were necessary and all items proved to build a single factor in the pilot phase.

**Table A.4. Development of the collegial nurse-physician relations scale**

| **Original item** | **Adapted item** | **Included in final survey** |
| --- | --- | --- |
| Physicians and nurses have good relationships. (44) | Physicians and nurses have good working relationships. | yes |
| A lot of teamwork between nurses and doctors. (44) | A lot of team work between nurses and physicians. | yes |
| Communication between nurses and physicians is open and positive. (45) | Communication between nurses and physicians on our unit is open and positive. | yes |
| When there is a disagreement between nurses and physicians, all points of view will be carefully considered in arriving at the best solutions to the problem. (45) | When there is a disagreement between nurses and physicians of our unit, all points of view will be carefully considered in arriving at the best solutions to the problem. | yes |
| Functional collaboration (joint practice) between nurses and physicians. (44) | Collaboration (joint patient care) between nurses and physicians is good. | yes |
| There is a good understanding of each other’s job responsibilities among all those involved in the care of patients in the NICU. (45) | Nurses and physicians of our unit have a good understanding of each other’s tasks and responsibilities. | yes |

Collaboration about care decisions: The original scale developed by Baggs had six items (46). Since the original scale refered to an individual dicision, we adapted the wording to represent decision-making processes in general, as had been done previouisly (47). Two of the items were repeatedly regarded as inappropriate or not comprehensible during the discussions with experts and the cognitive pretesting and were therefore excluded (see Table A.5.).

**Table A.5. Development of the collaboration about care decisions scale**

| **Original item** | **Adapted item** | **Included in final survey** |
| --- | --- | --- |
| Nurses and physicians planned together to make the decision about care for this patient. | Nurses and physicians plan together to make decisions about care for patients. | yes |
| Open commurucation between physicians and nurses took place as this decision was made for this patient. | Open communication between physicians and nurses takes place as decisions are made for patients. | yes |
| Decision-making responsibilities for this patient were shared between nurses and physicians. | Nurses and doctors share responsibility for decisions about patient care. | no ^a^ |
| Physicians and nurses cooperated in making this decision. | Physicians and nurses cooperate in making decisions regarding patient care. | yes |
| As this decision was considered, nurses and physicians each actively represented their professional perspectives about this patient's needs. | In making decisions about patient care, both nursing and medical concerns are considered. | yes |
| Decision makmg for this patient was co-ordinated between physicians and nurses. | Decision-making for patients is coordinated between doctors and nurses. | no ^b^ |

^a^ The item was deemed inappropriate because decision-making responsibility in the German healthcare system lies solely with doctors.

^b^ The item was rated as ambiguous and incomprehensible.

## Details on the development of the organizational questionnaire

In an iterative process involving discussions with experienced intensivists and critical care nurses, items were adapted from previously published items or new items were developed. The draft of the instrument was pretested by surveying six attending physicians working on two intensive care units of one university hospital. A final revision was done based on their written comments and discussions. Nurse autonomy in patient care was assessed by a newly developed instrument, which was based partly on items previously used in a nationwide survey of ICU head nurses in Germany (48).

# References

1. Hackman J, Oldham G. Development of the Job Diagnostic Survey. *Journal of Applied Psychology* 1975; 60: 159-170.

2. Stalpers D, Van Der Linden D, Kaljouw MJ, Schuurmans MJ. Nurse-perceived quality of care in intensive care units and associations with work environment characteristics: a multicentre survey study. *J Adv Nurs* 2017; 73: 1482-1490.

3. Chandrasekaran A, Mishra A. Task Design, Team Context, and Psychological Safety: An Empirical Analysis of R&D Projects in High Technology Organizations. *Production and Operations Management* 2012; 21: 977-996.

4. Pursio K, Kankkunen P, Sanner-Stiehr E, Kvist T. Professional autonomy in nursing: An integrative review. *J Nurs Manag* 2021; 29: 1565-1577.

5. Merriman C, Freeth D. Interprofessional ward rounds in an adult intensive care unit: an appreciative inquiry into the central collaboration between the consultant and the bedside nurse. *J Interprof Care* 2021: 1-9.

6. Braun JP, Kumpf O, Deja M, Brinkmann A, Marx G, Bloos F, Kaltwasser A, Dubb R, Muhl E, Greim C, Bause H, Weiler N, Chop I, Waydhas C, Spies C. The German quality indicators in intensive care medicine 2013--second edition. *Ger Med Sci* 2013; 11: Doc09.

7. DIVI. Peer Review Qualitätsindikatoren Intensivmedizin 4. Auflage 2022 2022.

8. Hillmann B, Schwarzkopf D, Manser T, Waydhas C, Riessen R. Structure and concept of ICU rounds: the VIS-ITS survey. *Med Klin Intensivmed Notfmed* 2022; 117: 276-282.

9. Nembhard IM, Edmondson AC. Making it safe: the effects of leader inclusiveness and professional status on psychological safety and improvement efforts in health care teams. *Journal of Organizational Behavior* 2006; 27: 941-966.

10. Hirak R, Peng AC, Carmeli A, Schaubroeck JM. Linking leader inclusiveness to work unit performance: The importance of psychological safety and learning from failures. *The Leadership Quarterly* 2012; 23: 107-117.

11. O'Donovan R, McAuliffe E. A systematic review of factors that enable psychological safety in healthcare teams. *Int J Qual Health Care* 2020; 32: 240-250.

12. Vazirani S, Hays RD, Shapiro MF, Cowan M. Effect of a multidisciplinary intervention on communication and collaboration among physicians and nurses. *Am J Crit Care* 2005; 14: 71-77.

13. LeTourneau B. Physicians and nurses: friends or foes? *J Healthc Manag* 2004; 49: 12-15.

14. Schmalenberg C, Kramer M. Nurse-physician relationships in hospitals: 20,000 nurses tell their story. *Crit Care Nurse* 2009; 29: 74-83.

15. Knaus W, Draper E, Wagner D, Zimmerman J. An evaluation of outcome from intensive care in major medical centers. *Can Crit Care Nurs J* 1987; 4: 15.

16. Carmeli A, Gittell JH. High-quality relationships, psychological safety, and learning from failures in work organizations. *Journal of Organizational Behavior* 2009; 30: 709-729.

17. Pearce CL, Herbik PA. Citizenship Behavior at the Team Level of Analysis: The Effects of Team Leadership, Team Commitment, Perceived Team Support, and Team Size. *The Journal of Social Psychology* 2004; 144: 293-310.

18. re:Work. Guide: Understand team effectiveness. [cited 2023 09.01.2023]. Available from: <https://rework.withgoogle.com/print/guides/5721312655835136/>.

19. Valentine MA, Edmondson AC. Team Scaffolds: How Mesolevel Structures Enable Role-Based Coordination in Temporary Groups. *Organization Science* 2015; 26: 405-422.

20. Midthaug M. The Relationship between Management Team Size and Team Performance: The Mediating Effect of Team Psychological Safety. 2017.

21. Schwappach DL, Gehring K. Trade-offs between voice and silence: a qualitative exploration of oncology staff's decisions to speak up about safety concerns. *BMC Health Serv Res* 2014; 14: 303.

22. Remtulla R, Hagana A, Houbby N, Ruparell K, Aojula N, Menon A, Thavarajasingam SG, Meyer E. Exploring the barriers and facilitators of psychological safety in primary care teams: a qualitative study. *BMC Health Services Research* 2021; 21: 269.

23. Dunbar RIM. Coevolution of neocortical size, group size and language in humans. *Behavioral and Brain Sciences* 1993; 16: 681-694.

24. Lindenfors P, Wartel A, Lind J. 'Dunbar's number' deconstructed. *Biol Lett* 2021; 17: 20210158.

25. de Ruiter J, Weston G, Lyon SM. Dunbar's number: group size and brain physiology in humans reexamined. *Am Anthropol* 2011; 113: 557-568.

26. Freeberg TM, Dunbar RI, Ord TJ. Social complexity as a proximate and ultimate factor in communicative complexity. *Philos Trans R Soc Lond B Biol Sci* 2012; 367: 1785-1801.

27. Psychological Safety 82: Dunbar’s Number and Team Size. 2022 [cited 2023 09.01.2023]. Available from: <https://psychsafety.co.uk/psychological-safety-82-dunbars-number-and-team-size/>.

28. Meyer JP, Allen NJ. A three-component conceptualization of organizational commitment. *Human Resource Management Review* 1991; 1: 61-89.

29. Brief AP, Weiss HM. Organizational Behavior: Affect in the Workplace. *Annual Review of Psychology* 2002; 53: 279-307.

30. Judge TA, Thoresen CJ, Bono JE, Patton GK. The job satisfaction-job performance relationship: A qualitative and quantitative review. *Psychol Bull* 2001; 127: 376-407.

31. Meyer JP, Stanley DJ, Herscovitch L, Topolnytsky L. Affective, continuance, and normative commitment to the organization: A meta-analysis of antecedents, correlates, and consequences. *J Vocat Behav* 2002; 61: 20-52.

32. Galletta M, Portoghese I, Carta MG, D'Aloja E, Campagna M. The Effect of Nurse-Physician Collaboration on Job Satisfaction, Team Commitment, and Turnover Intention in Nurses. *Res Nurs Health* 2016; 39: 375-385.

33. Frazier ML, Fainshmidt S, Klinger RL, Pezeshkan A, Vracheva V. Psychological Safety: A Meta-Analytic Review and Extension. *Personnel Psychology* 2017; 70: 113-165.

34. Li J, Li S, Jing T, Bai M, Zhang Z, Liang H. Psychological Safety and Affective Commitment Among Chinese Hospital Staff: The Mediating Roles of Job Satisfaction and Job Burnout. *Psychol Res Behav Manag* 2022; 15: 1573-1585.

35. Herscovitch L, Meyer JP. Commitment to organizational change: Extension of a three-component model. *J Appl Psychol* 2002; 87: 474-487.

36. Nembhard IM, Alexander JA, Hoff TJ, Ramanujam R. Why Does the Quality of Health Care Continue to Lag? Insights from Management Research. *Acad Manag Perspect* 2009; 23: 24-42.

37. Bloos F, Rüddel H, Thomas-Rüddel D, Schwarzkopf D, Pausch C, Harbarth S, Schreiber T, Gründling M, Marshall J, Simon P, Levy MM, Weiss M, Weyland A, Gerlach H, Schürholz T, Engel C, Matthäus-Krämer C, Scheer C, Bach F, Riessen R, Poidinger B, Dey K, Weiler N, Meier-Hellmann A, Häberle HH, Wöbker G, Kaisers UX, Reinhart K. Effect of a multifaceted educational intervention for anti-infectious measures on sepsis mortality: a cluster randomized trial. *Intensive Care Med* 2017; 43: 1602 - 1612.

38. Schwarzkopf D, Matthaeus-Kraemer CT, Thomas-Rüddel DO, Rüddel H, Poidinger B, Bach F, Gerlach H, Gründling M, Lindner M, Scheer C, Simon P, Weiss M, Reinhart K, Bloos F. A multifaceted educational intervention improved anti-infectious measures but had no effect on mortality in patients with severe sepsis. *Scientific Reports* 2022; 12: 3925.

39. Tucker AL, Nembhard IM, Edmondson AC. Implementing new practices: An empirical study of organizational learning in hospital intensive care units. *Manage Sci* 2007; 53: 894-907.

40. Edmondson A. Psychological safety and learning behavior in work teams. *Adm Sci Q* 1999; 44: 350-383.

41. Felfe J, Six B, Schmook R, Knorz C. Commitment organization, job and form of employment [Commitment Organisation, Beruf und Beschäftigungsform (COBB)]. Zusammenstellung sozialwissenschaftlicher Items und Skalen (ZIS); 2002.

42. Schwarzkopf D, Rüddel H, Thomas-Rüddel DO, Felfe J, Poidinger B, Matthäus-Krämer CT, Hartog CS, Bloos F. Perceived nonbeneficial treatment of patients, burnout, and intention to leave the job among ICU nurses and junior and senior physicians. *Crit Care Med* 2017; 45: e265-e273.

43. Herrmann D, Felfe J, Hardt J. Transformational leadership and readiness for change: stressors and resources as key contextual factors [Transformationale Führung und Veränderungsbereitschaft. Stressoren und Ressourcen als relevante Kontextbedingungen]. *Zeitschrift für Arbeits- und Organisationspsychologie* 2012; 56: 70-86.

44. Lake ET. Development of the practice environment scale of the Nursing Work Index. *Res Nurs Health* 2002; 25: 176-188.

45. Nembhard IM, Tucker AL. Deliberate learning to improve performance in dynamic service settings: Evidence from hospital intensive care units. *Organ Sci* 2011; 22: 907-922.

46. Baggs JG. Development of an instrument to measure collaboration and satisfaction about care decisions. *J Adv Nurs* 1994; 20: 176-182.

47. Maxson PM, Dozois EJ, Holubar SD, Wrobleski DM, Dube JAO, Klipfel JM, Arnold JJ. Enhancing nurse and physician collaboration in clinical decision making through high-fidelity interdisciplinary simulation training. *Mayo Clin Proc* 2011; 86: 31-36.

48. Isfort M, Weidner F, Gehlen D. Nursing thermometer 2012: a nationwide survey of nursing leaders on the situation of nursing care and patient care in intensive care units in hospitals [Pflege-Thermometer 2012. Eine bundesweite Befragung von Leitungskräften zur Situation der Pflege und Patientenversorgung auf Intensivstationen im Krankenhaus]. [PDF] 2012 [cited 2026 April 13th]. Available from: <https://www.dip.de/wp-content/uploads/2025/02/Pflege_Thermometer_2012.pdf>.

1. In the German system, ward physicians (“Stationsärzte”) comprise both residents and board-certified physicians working in non-supervisory roles, and are distinct from senior physicians (“Oberärzte”). In this survey, ward physicians were addressed as a single group, irrespective of training level. [↑](#footnote-ref-1)
