# Supplementary material for: Designing Nurse–Physician Collaboration to Improve Psychological Safety, Satisfaction and Commitment of Critical Care Nurses—A Multi‐Informant Survey Study
Source: Nurs Crit Care. 2026 Jul 1;31(4):e70567. doi: 10.1111/nicc.70567 (PMC13320614; doi:10.1111/nicc.70567)
Supplement: Supplementary file 3 — Supporting Information: C. A translation of the centre level questionnaire. [file NICC-31-0-s004.docx]

Supplementary Material C:

Center-level questionnaire for intensive care units

In the following, you will be asked to provide information about your hospital and your intensive care unit. The questionnaire should be completed by the medical director of the ICU or a senior ICU physician with several years of experience on the respective unit. For some items, it may be advisable to consult the nurse manager of the ICU. All data will be stored in pseudonymized form (using a code number). They will be treated in strict confidence, accessed only by members of the MEDUSA study team, and not made available to third parties. Any reports based on the collected data that are shared with third parties will be presented in such a way that no conclusions can be drawn about the identity of your institution (only aggregated statistics across groups of hospitals or units will be reported).

**Hospital characteristics**

| Category | ○ university hospital ○ teaching hospital ○ general hospital |
| --- | --- |
| Ownership | ○ public ○ non-profit ○ private |
| Level of care | ○ primary care ○ secondary care  ○ tertiary care |
| Number of hospital beds | _ _ _ _ |

**Information on the hospital’s intensive care units**

Please list all intensive care units in the hospital, along with the department or departments responsible for each unit. Please begin with your own intensive care unit.

| **Name of the ICU** | **Department(s) responsible for the ICU** |
| --- | --- |
| Your ICU: |  |
| _____________________ | _____________________ |
| Other ICUs: |  |
| _____________________ | _____________________ |
| _____________________ | _____________________ |
| _____________________ | _____________________ |
| _____________________ | _____________________ |

Detailed information on the intensive care unit

Please provide the following information about your intensive care unit. If the ICU is managed by multiple departments, please base your responses on the beds cared for by your department.

**Allgemeine Angaben**

| Number of ICU beds | _ _ _ |
| --- | --- |
| Number of ventilated beds | _ _ _ |
| Number of cases treated per year | _ _ _ _ _ |

**Information on ICU staff**

Please provide the following information on personnel currently working regularly in your ICU.

| **Berufs/Hierarchiegruppe** | **Number of persons** |
| --- | --- |
| Senior physicians | _ _ |
| Of these, with additional qualification in intensive care medicine | _ _ |
| Ward physicians | _ _ |
| Of these, board-certified physicians | _ _ |
| Of these, with additional qualification in intensive care medicine | _ _ |
| Nurse managers (unit level) | _ _ |
| Is there an additional supervisory level below the nurse managers (e.g., “team leader”)?  ⭘ yes ⭘ no |  |
| If yes, how many individuals? | _ _ |
| Nurses with regular shift leader responsibilities | _ _ |
| Nurses without supervisory roles | _ _ |
| Of these, with completed specialist training in intensive care | _ _ |
| Occupational / physical therapists (assigned to the ICU or affiliated with your department) | _ _ |
| Psychologists (assigned to the ICU or affiliated with your department) | _ _ |
| Social workers / social pedagogues (assigned to the ICU or affiliated with your department) | _ _ |

**Autonomy of nursing staff**Below is a list of therapeutic interventions. Please estimate to what extent nurses in your ICU perform these interventions independently. Base your assessment on what typically applies to sufficiently experienced nurses in your ICU.

|  | Nurse determines necessity of the treatment and conducts it autonomously (with possible subsequent notification of a physician) | Nurse conducts treatment autonomously after physician's prescription | Treatment is only conducted by physician or under physician's direct control (physician present) |
| --- | --- | --- | --- |
| Laboratory testing (blood gas analysis) | ⭘ | ⭘ | ⭘ |
| Insertion of new indwelling venous cannula | ⭘ | ⭘ | ⭘ |
| Insertion of new indwelling bladder catheter | ⭘ | ⭘ | ⭘ |
| Changing dressings of central venous catheter | ⭘ | ⭘ | ⭘ |
| Changing dressings of respiration tube | ⭘ | ⭘ | ⭘ |
| Conduction of weaning from ventilation | ⭘ | ⭘ | ⭘ |

|  | Initiation of therapy | Adjustment of an ongoing therapy | | |
| --- | --- | --- | --- | --- |
|  | Nurse determines the necessity of the treatment and initiates it autonomously (with possible subsequent notification of a physician) | Nurse determines necessity of adjustment of treatment and conducts it autonomously (with possible subsequent notification of a physician) | Physician sets concrete target values for parameters and nurse adjust the therapy to achieve the target values | Adjustment of therapy is only done after concrete order of physician or by the physician |
| Adjustment of oxygen concentration during ventilation | _ | ⭘ | ⭘ | ⭘ |
| Adjustment of peak pressure and breathing rate during ventilation | _ | ⭘ | ⭘ | ⭘ |
| Adjustment of positive end-expiratory pressure during ventilation | _ | ⭘ | ⭘ | ⭘ |
| Adjustment of calcium and citrate during dialysis | _ | ⭘ | ⭘ | ⭘ |
| Catecholamine therapy | ⭘ | ⭘ | ⭘ | ⭘ |
| Control of sedation | ⭘ | ⭘ | ⭘ | ⭘ |
| Control of blood glucose through administration of insulin | ⭘ | ⭘ | ⭘ | ⭘ |
| Administration of analgesics | ⭘ | ⭘ | ⭘ | ⭘ |
| Intravenous administration of antihypertensive agents | ⭘ | ⭘ | ⭘ | ⭘ |
| Administration of crystalloid infusions | ⭘ | ⭘ | ⭘ | ⭘ |
| Administration of caogulation factors | ⭘ | ⭘ | ⭘ | ⭘ |
| Control of potassium at the syringe pump | ⭘ | ⭘ | ⭘ | ⭘ |
| Administration of oxygen via nasal tube or mask | ⭘ | ⭘ | ⭘ | ⭘ |

**Participation in ward rounds**

The following section addresses how frequently different professional and hierarchical groups participate in ward rounds. Participation is defined as active involvement, not merely being present in the room, and implies pausing other duties to take part exclusively in the ward round.

|  | Always | Often | Sometimes | Seldom | Never |
| --- | --- | --- | --- | --- | --- |
| ICU physician ward round |  |  |  |  |  |
| Participation of the responsible ward physician | ⭘ | ⭘ | ⭘ | ⭘ | ⭘ |
| Participation of the responsible senior physician | ⭘ | ⭘ | ⭘ | ⭘ | ⭘ |
| Participation of the head of department / ICU director | ⭘ | ⭘ | ⭘ | ⭘ | ⭘ |
| Participation of the responsible nurse | ⭘ | ⭘ | ⭘ | ⭘ | ⭘ |
| Participation of the nurse manager (unit level) | ⭘ | ⭘ | ⭘ | ⭘ | ⭘ |
| Joint physician ward rounds with the primary admitting service^[[1]](#footnote-1)^  Do such ward rounds take place in your ICU?: ⭘ yes ⭘ no  If yes: |  | | | | |
| Participation of the responsible ward physician | ⭘ | ⭘ | ⭘ | ⭘ | ⭘ |
| Participation of the responsible senior physician | ⭘ | ⭘ | ⭘ | ⭘ | ⭘ |
| Participation of the head of department / ICU director | ⭘ | ⭘ | ⭘ | ⭘ | ⭘ |
| Participation of the responsible nurse | ⭘ | ⭘ | ⭘ | ⭘ | ⭘ |
| Participation of the nurse manager (unit level) | ⭘ | ⭘ | ⭘ | ⭘ | ⭘ |

1. i.e., the admitting specialty responsible for the patient, e.g., surgery [↑](#footnote-ref-1)
