# Supplementary material for: Designing Nurse–Physician Collaboration to Improve Psychological Safety, Satisfaction and Commitment of Critical Care Nurses—A Multi‐Informant Survey Study
Source: Nurs Crit Care. 2026 Jul 1;31(4):e70567. doi: 10.1111/nicc.70567 (PMC13320614; doi:10.1111/nicc.70567)
Supplement: Supplementary file 4 — Supporting Information D. Supplemental Figures and Tables. Figure S1: Study flow chart. Response rate defined as number of returned questionnaires divided by number of staff reported by local study coordinators. Table S1: Characteristics of participating hospitals and intensive care units. Table S2: Characteristics of participating physicians. Table S3: Items of the survey of nurses. Table S4: Items of the survey of physicians. Table S5: Factor loadings of confirmatory factor analysis of items of survey of nurses. Table S6: Factor loadings of confirmatory factor analysis of items of survey of physicians. Table S7: Distribution of scale scores, internal consistency and interrater reliability. Table S8: Descriptive statistics and correlations for study measures aggregated to the unit level. [file NICC-31-0-s002.docx]

Supplementary Material D – Supplementary Figures and Tables


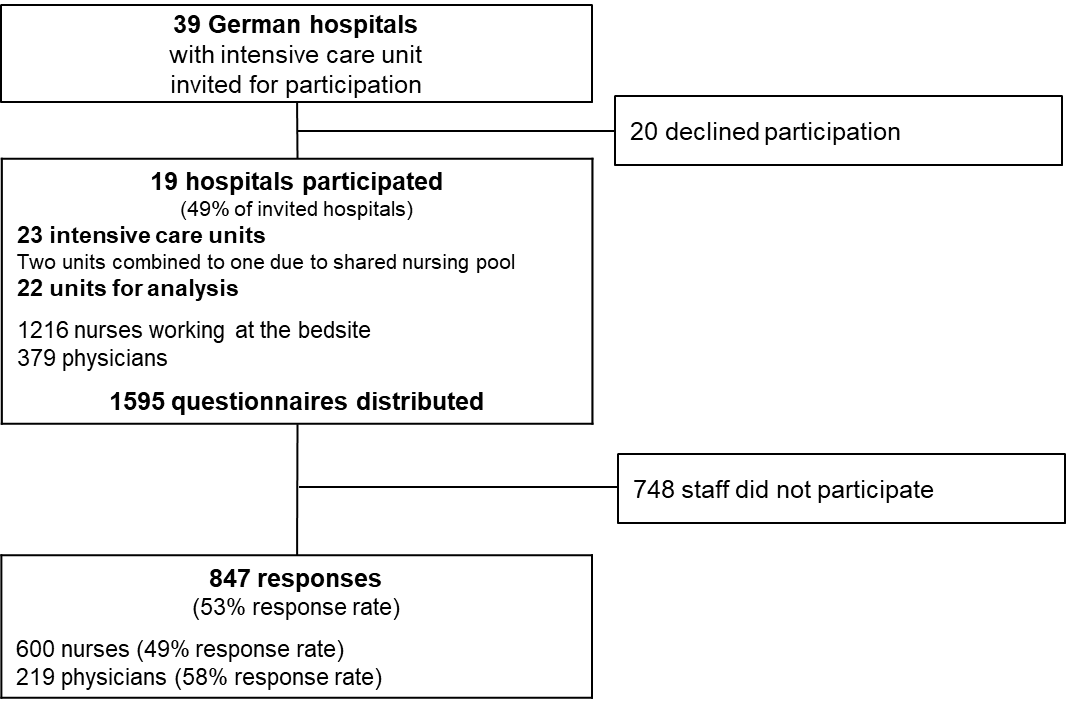


**Figure S1. Study flow chart**Response rate defined as number of returned questionnaires divided by number of staff reported by local study coordinators.

**Table S1. Characteristics of participating hospitals and intensive care units**

| **Variables** | **Descriptive statistics** |
| --- | --- |
| **Hospital characteristics (N = 19)** |  |
| University hospital | 6 (31.6%) |
| Level of care: Primary care hospital | 7 (36.8%) |
| Secondary care hospital | 3 (15.8%) |
| Tertiary care hospital | 9 (47.4%) |
| Hospital operator: Public | 12 (63.2%) |
| Non-profit | 3 (15.8%) |
| Private | 4 (21.1%) |
| Number of hospital beds | 800 [330, 1312] |
| **ICU characteristics (N = 22)** |  |
| Type of unit: Medical ICU | 5 (22.7%) |
| Mixed ICU | 9 (40.9%) |
| Neurological ICU | 1 (4.5%) |
| Surgical ICU | 7 (31.8%) |
| Number of ICU beds | 12 [9.25, 18.25] |
| Number of nurses per unit | 37.5 [26.25, 75] |
| Number of nurses per unit participating in survey | 19 [13, 34.5] |
| Proportion of nurses per unit participating in survey (%) | 50.84 [47.66, 61.01] |
| Participation of nurses in patient rounds: Always | 10 (45.5%) |
| Often | 10 (45.5%) |
| Sometimes | 1 (4.5%) |
| Seldom | 1 (4.5%) |
| Never | 0 (0%) |

Descriptive characteristics given as N (%) or median [1^st^ quartile, 3^rd^ quartile]. ICU: intensive care unit

**Table S2. Characteristics of participating physicians**

| **Variable** | **N (non-missing)** | **Descriptive statistics** |
| --- | --- | --- |
| Age: < 30 | 210 | 26 (12.4%) |
| 30 - 39 |  | 109 (51.9%) |
| 40 - 49 |  | 50 (23.8%) |
| >= 50 |  | 25 (11.9%) |
| Sex: female | 210 | 97 (46.2%) |
| Senior attending physician: yes^a^ | 219 | 70 (32%) |
| Experience as a physician (years): < 1 | 204 | 8 (3.9%) |
| 1 - 2 |  | 11 (5.4%) |
| 3 - 5 |  | 56 (27.5%) |
| 6 - 10 |  | 46 (22.5%) |
| > 10 |  | 83 (40.7%) |
| ICU experience (years): < 1 | 209 | 56 (26.8%) |
| 1 - 2 |  | 39 (18.7%) |
| 3 - 5 |  | 36 (17.2%) |
| 6 - 10 |  | 28 (13.4%) |
| > 10 |  | 50 (23.9%) |
| Critical care specialization | 195 | 43 (22.1%) |
| Working full time on the ICU | 184 | 87 (47.3%) |
| Working hours per week | 195 | 50 [45, 55] |
| Working nights per month | 193 | 5 [3, 6] |
| Weekend work days per month | 195 | 3 [2, 4] |

Descriptive statistics for N = 219 participating physicians, given as N (%) or median [1^st^ quartile, 3^rd^ quartile].

^a^ This corresponds to the position “Oberarzt”, which is a senior ICU physician supervising residents and fellows, managing care, with significant clinical leadership responsibilities.

**Table S3. Items of the survey of nurses**

| **Items** | **N (non missing)** | **Minimum** | **Maximum** | **Mean±SD** |
| --- | --- | --- | --- | --- |
| **Psychological safety** |  |  |  |  |
| 1. Nurses in this unit are comfortable checking with other nurses or physicians of the team if they have questions about the right way to do something. | 595 | 1 | 7 | 5±1.2 |
| 2. The nurses in this unit are able to bring up problems and tough issues. | 594 | 1 | 7 | 4.4±1.4 |
| 3. Nurses who state a differing opinion are often confronted with rejection in this unit. | 594 | 1 | 7 | 4.2±1.4 |
| 4. If a nurse makes a mistake in this unit, it is often held against him. | 592 | 1 | 7 | 3.5±1.5 |
| 5. For nurses in this unit it is difficult to ask other nurses or physicians of the team for help. | 592 | 1 | 7 | 3.1±1.2 |
| **Affective commitment to the unit** |  |  |  |  |
| 1. I am proud to belong to this unit. | 596 | 1 | 7 | 5±1.6 |
| 2. I feel a strong sense of belonging to my unit. | 595 | 1 | 7 | 4.8±1.6 |
| 3. I think that my moral values fit those of the unit. | 597 | 1 | 7 | 4.4±1.6 |
| 4. I don’t feel particularly emotionally attached to this unit. | 595 | 1 | 7 | 3.1±1.7 |
| **Commitment to change** |  |  |  |  |
| 1. I believe that we are actively approaching changes in working processes to improve patient care instead of just reacting, if necessary. | 594 | 1 | 7 | 4±1.4 |
| 2. I believe there will be changes of working processes in our unit that will improve the quality of patient care in the next years. | 591 | 1 | 7 | 4±1.6 |
| 3. I think that I have no possibility to influence changes in working processes for the improvement of patient care on this unit. | 595 | 1 | 7 | 4±1.7 |
| **Job Satisfaction** |  |  |  |  |
| 1. Overall, how satisfied are you with your job on your unit? | 594 | 1 | 7 | 4.2±1.4 |

SD: Standard deviation.

**Table S4. Items of the survey of physicians**

| **Items** | **N (non missing)** | **Minimum** | **Maximum** | **Mean±SD** |
| --- | --- | --- | --- | --- |
| **Collegial nurse-physician relations** |  |  |  |  |
| 1. Physicians and nurses have good working relationships | 217 | 2 | 7 | 5.2±1.1 |
| 2. A lot of team work between nurses and physicians | 216 | 2 | 7 | 5.2±1.1 |
| 3. Communication between nurses and physicians on our unit is open and positive. | 216 | 2 | 7 | 5.1±1.1 |
| 4. When there is a disagreement between nurses and physicians of our unit, all points of view will be carefully considered in arriving at the best solutions to the problem. | 217 | 2 | 7 | 4.5±1.2 |
| 5. Collaboration (joint patient care) between nurses and physicians is good | 216 | 2 | 7 | 5.3±1.1 |
| 6. Nurses and physicians of our unit have a good understanding of each other’s tasks and responsibilities. | 216 | 2 | 7 | 4.9±1.2 |
| **Collaboration about care-decisions** |  |  |  |  |
| 1. Nurses and physicians plan together to make decisions about care for patients. | 217 | 1 | 7 | 4.4±1.3 |
| 2. Open communication between physicians and nurses takes place as decisions are made for patients. | 217 | 1 | 7 | 4.9±1.2 |
| 3. Physicians and nurses cooperate in making decisions regarding patient care. | 216 | 2 | 7 | 4.9±1.1 |
| 4. In making decisions about patient care, both nursing and medical concerns are considered. | 216 | 2 | 7 | 5.1±1.1 |

SD: Standard deviation.

**Table S5. Factor loadings of confirmatory factor analysis of items of survey of nurses.**

| **Latent factor** | **Item** | **Loading (95% CI)** | **P-value** |
| --- | --- | --- | --- |
| Psychological safety | Item_01 | 1 (1, 1) |  |
| Psychological safety | Item_02 | 1.33 (1.15, 1.51) | <=0.001 |
| Psychological safety | Item_03 | -1.27 (-1.47, -1.08) | <=0.001 |
| Psychological safety | Item_04 | -1.18 (-1.38, -0.98) | <=0.001 |
| Psychological safety | Item_05 | -0.82 (-0.98, -0.67) | <=0.001 |
| Commitment to change | Item_01 | 1 (1, 1) |  |
| Commitment to change | Item_02 | 1.21 (1.02, 1.39) | <=0.001 |
| Commitment to change | Item_03 | -1.31 (-1.51, -1.1) | <=0.001 |
| Affective commitment to the unit | Item_01 | 1 (1, 1) |  |
| Affective commitment to the unit | Item_02 | 1.05 (0.98, 1.11) | <=0.001 |
| Affective commitment to the unit | Item_03 | 0.96 (0.89, 1.02) | <=0.001 |
| Affective commitment to the unit | Item_04 | -0.85 (-0.94, -0.77) | <=0.001 |
| Job satisfaction | Item_01 | 1 (1, 1) |  |

Results based on confirmatory factor analysis of survey responses of N = 600 nurses using full information maximum likelihood estimation. CI: Confidence interval. Item wordings are presented in Table S3.

**Table S6. Factor loadings of confirmatory factor analysis of items of survey of physicians.**

| Latent factor | Item | Loading | P.value |
| --- | --- | --- | --- |
| Collaboration about care-decisions | Item_01 | 1 (1, 1) |  |
| Collaboration about care-decisions | Item_02 | 0.99 (0.83, 1.14) | <=0.001 |
| Collaboration about care-decisions | Item_03 | 0.86 (0.72, 1) | <=0.001 |
| Collaboration about care-decisions | Item_04 | 0.93 (0.78, 1.08) | <=0.001 |
| Collegial nurse-physician relations | Item_01 | 1 (1, 1) |  |
| Collegial nurse-physician relations | Item_02 | 1.1 (0.93, 1.27) | <=0.001 |
| Collegial nurse-physician relations | Item_03 | 1.15 (0.98, 1.33) | <=0.001 |
| Collegial nurse-physician relations | Item_04 | 1.19 (0.98, 1.4) | <=0.001 |
| Collegial nurse-physician relations | Item_05 | 1.11 (0.94, 1.28) | <=0.001 |
| Collegial nurse-physician relations | Item_06 | 1.1 (0.91, 1.29) | <=0.001 |

Results based on confirmatory factor analysis of survey responses of N = 217 physicians using full information maximum likelihood estimation. CI: Confidence interval. Item wordings are presented in Table S4.

**Table S7. Distribution of scale scores, internal consistency and interrater reliablity**

| **Scale** | **N (non missing)** | **Mean**±**SD** | **Minimum** | **Maximum** | **Cronbachs alpha** | **ICC1** | **ICC2** |
| --- | --- | --- | --- | --- | --- | --- | --- |
| **Physician survey** |  |  |  |  |  |  |  |
| Collegial nurse-physician relations | 217 | 5±0.9 | 2,17 | 7 | 0.91 | 0,15 | 0,64 |
| Collaboration about care-decisions | 217 | 4.8±1 | 1,75 | 7 | 0.88 | 0,19 | 0,7 |
| Mean of the two scales^a^ | 217 | 4.9±0.9 | 2,12 | 7 | - | 0,2 | 0,72 |
| **Nurse survey** |  |  |  |  |  |  |  |
| Psychological safety | 596 | 4.5±1 | 1 | 7 | 0.78 | 0,21 | 0,88 |
| Affective commitment to the unit | 599 | 4.8±1.4 | 1 | 7 | 0.9 | 0,15 | 0,83 |
| Commitment to change | 596 | 4±1.2 | 1 | 7 | 0.71 | 0,09 | 0,73 |
| Job Satisfaction^b^ | 594 | 4.2±1.4 | 1 | 7 | - | 0,15 | 0,83 |

ICC: intraclass correlation. ICC1 represents the proportion of the variance in individual ratings, which can be explained by group membership (the intensive care unit), ICC2 represents the reliability of the means at the unit level and depends both on ICC1 as well as the group size (number of staff per unit).

^a^ Further analyses were based on the mean of both scales (per physician), since both scales showed a relatively high Pearson correlation (r = 0.67).

^b^ Job satisfaction was measured by one item.

**Table S8. Descriptive statistics and correlations for study measures aggregated to the unit level.**

|  | Descriptives | 1 | 2 | 3 | 4 | 5 | 6 | 7 | 8 |
| --- | --- | --- | --- | --- | --- | --- | --- | --- | --- |
| 1. Number of nurses working on unit^a^ | 55.3±43.5 | 1 |  |  |  |  |  |  |  |
| 2. Nurses always participating in rounds^a^ | 10 (45.5%) | -0.41 | 1 |  |  |  |  |  |  |
| 3. Nurses’ autonomy in patient care^a^ | 0±0.6 | -0.29 | 0.44* | 1 |  |  |  |  |  |
| 4. Quality of nurse-physician relations^b^ | 4.9±0.5 | -0.51* | 0.4 | 0.51* | 1 |  |  |  |  |
| 5. Psychological safety^c^ | 4.7±0.5 | -0.55** | 0.51* | 0.51* | 0.59** | 1 |  |  |  |
| 6. Affective commitment^c^ | 5.1±0.6 | -0.71*** | 0.51* | 0.25 | 0.49* | 0.82*** | 1 |  |  |
| 7. Job satisfaction^c^ | 4.4±0.6 | -0.47* | 0.51* | 0.28 | 0.55** | 0.64** | 0.66*** | 1 |  |
| 8. Commitment to change^c^ | 4.2±0.5 | -0.62** | 0.65*** | 0.58** | 0.59** | 0.84*** | 0.86*** | 0.75*** | 1 |

Statistics given for measures aggregated to the unit level for N = 22 intensive care units (ICUs). Descriptive statistics presented as N (%) or Mean±SD (SD: standard deviation). Significance of Pearson correlation: *** ≤0.001, ** >0.001 & ≤0.01, * >0.01 & ≤0.05.
^a^ Measure obtained by survey of leading intensivist of the ICU
^b^ Measure obtained by survey of ICU physicians.
^c^ Measure obtained by survey of ICU nurses.
